# Congenital idiopathic megaesophagus in the German shepherd dog is a sex-differentiated trait and is associated with an intronic variable number tandem repeat in *Melanin-Concentrating Hormone Receptor 2*

**DOI:** 10.1371/journal.pgen.1010044

**Published:** 2022-03-10

**Authors:** Sarah M. Bell, Jacquelyn M. Evans, Katy M. Evans, Kate L. Tsai, Rooksana E. Noorai, Thomas R. Famula, Dolores M. Holle, Leigh Anne Clark

**Affiliations:** 1 Department of Genetics and Biochemistry, Clemson University, Clemson, South Carolina, United States of America; 2 Cancer Genetics and Comparative Genomics Branch, National Human Genome Research Institute, National Institutes of Health, Bethesda, Maryland, United States of America; 3 The Seeing Eye Inc., Morristown, New Jersey, United States of America; 4 School of Veterinary Medicine and Science, University of Nottingham, Sutton Bonington, United Kingdom; 5 Clemson University Genomics and Bioinformatics Facility, Clemson University, Clemson, South Carolina, United States of America; 6 Department of Animal Science, University of California, Davis, California, United States of America; HudsonAlpha Institute for Biotechnology, UNITED STATES

## Abstract

Congenital idiopathic megaesophagus (CIM) is a gastrointestinal (GI) motility disorder of dogs in which reduced peristaltic activity and dilation of the esophagus prevent the normal transport of food into the stomach. Affected puppies regurgitate meals and water, fail to thrive, and experience complications such as aspiration pneumonia that may necessitate euthanasia. The German shepherd dog (GSD) has the highest disease incidence, indicative of a genetic predisposition. Here, we discover that male GSDs are twice as likely to be affected as females and show that the sex bias is independent of body size. We propose that female endogenous factors (*e*.*g*., estrogen) are protective via their role in promoting relaxation of the sphincter between the esophagus and stomach, facilitating food passage. A genome-wide association study for CIM revealed an association on canine chromosome 12 (*P*-val = 3.12x10^-13^), with the lead SNPs located upstream or within *Melanin-Concentrating Hormone Receptor 2* (*MCHR2*), a compelling positional candidate gene having a role in appetite, weight, and GI motility. Within the first intron of *MCHR2*, we identified a 33 bp variable number tandem repeat (VNTR) containing a consensus binding sequence for the T-box family of transcription factors. Across dogs and wolves, the major allele includes two copies of the repeat, whereas the predominant alleles in GSDs have one or three copies. The single-copy allele is strongly associated with CIM (*P*-val = 1.32x10^-17^), with homozygosity for this allele posing the most significant risk. Our findings suggest that the number of T-box protein binding motifs may correlate with *MCHR2* expression and that an imbalance of melanin-concentrating hormone plays a role in CIM. We describe herein the first genetic factors identified in CIM: sex and a major locus on chromosome 12, which together predict disease state in the GSD with greater than 75% accuracy.

## Author summary

German shepherd dogs (GSDs) are predisposed to an inherited motility disorder of the esophagus, termed congenital idiopathic megaesophagus (CIM), in which swallowing is ineffective and the esophagus is enlarged. Affected puppies are unable to properly pass food into their stomachs and consequently regurgitate their meals and show a failure to thrive, often leading to euthanasia. Here, we discovered that male GSDs are affected at a ratio of almost 2-to-1 over females, suggesting a protective biological advantage in females. In humans, estrogen is thought to play a role in the male predominance of esophageal disorders like reflux esophagitis and esophageal cancer. In a genome-wide scan, we identified an association with CIM on chromosome 12 and, within this region, a repetitive sequence in *MCHR2*. This gene encodes a receptor for melanin-concentrating hormone, a signaling molecule that is linked to appetite, weight, and gut motility. Together, sex and the *MCHR2* repeat sequence accurately predict affection status in over 75% of dogs, and a genetic test is now available to facilitate breeding decisions aimed at reducing disease incidence.

## Introduction

Esophageal motility is an integrated neuromuscular process that, when dysregulated, causes an array of digestive disturbances [1]. Normally, the consumption of foods and liquids stimulates afferent signaling of vagus nerve receptors extending from the pharynx to the lower esophageal sphincter (LES), triggering an efferent vagal response comprised of peristaltic contractions and LES relaxation [2,3]. In humans, the most recognized and studied esophageal dysmotility is achalasia [4], characterized by constriction of the LES and aperistalsis, causing difficulty swallowing, coughing, chest pain, and regurgitation [5,6].

**Fig 1:**
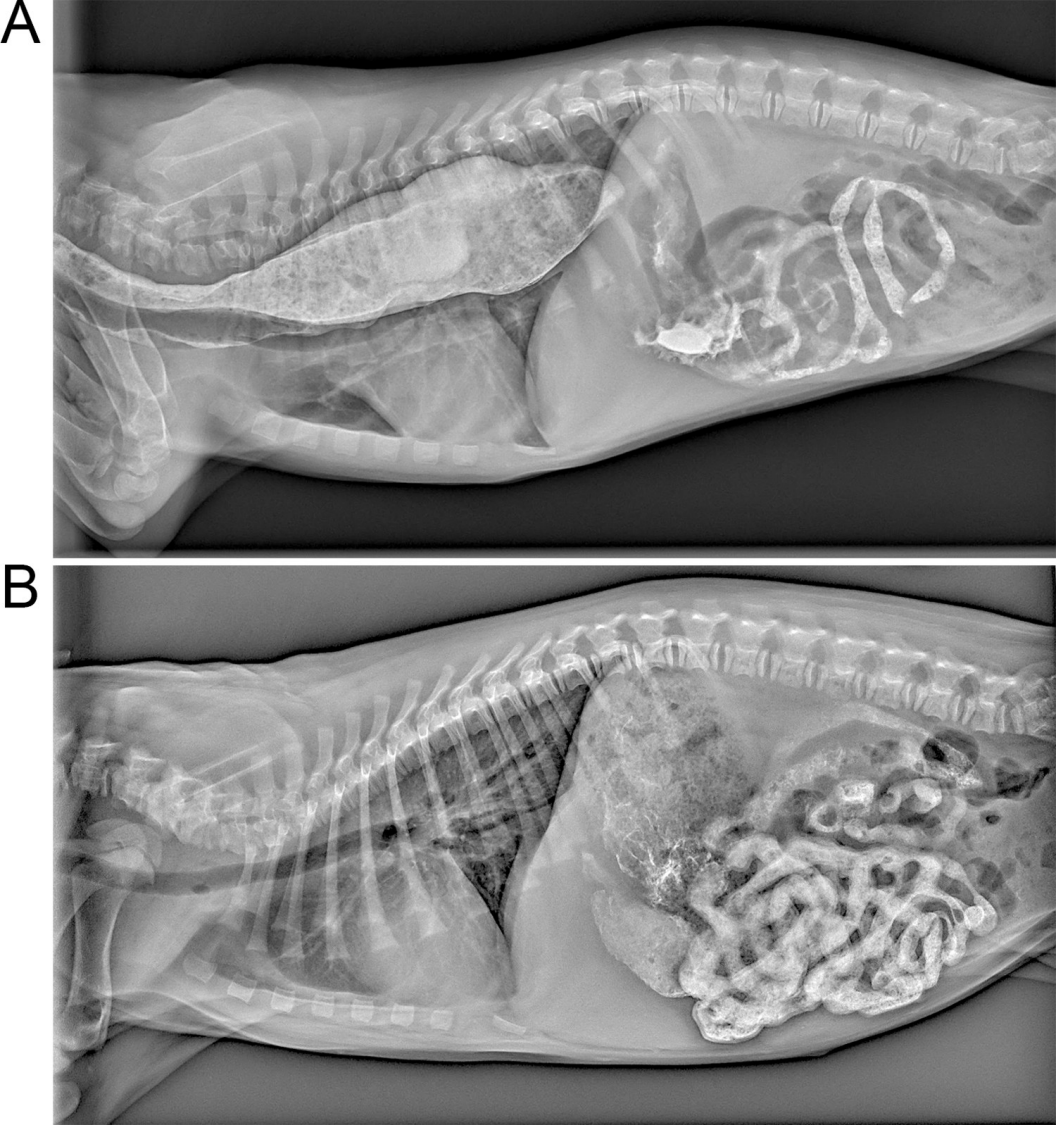
Barium-contrast radiographs of affected and healthy puppies. Radiographs were taken 40 minutes after a barium meal in five-week old puppies. A) Barium coats the enlarged esophagus of a CIM-affected GSD and (B) passes through to the stomach and intestines in a healthy GSD.

The most common esophageal dysmotility in dogs is congenital idiopathic megaesophagus (CIM) [7]. While gravity aids motility of the vertical human esophagus, it does not facilitate food passage in the horizontally-oriented canine esophagus. CIM-affected dogs have ineffective peristalsis, which leads to food retention that stretches and dilates the esophagus [8]. Overt clinical signs include coughing and regurgitation, usually beginning upon weaning at around four weeks of age [3,9]. CIM encompasses a broad phenotypic spectrum ranging from subclinical cases that may only be detected via radiography to severe cases with regurgitation episodes several times a day [7,9]. A CIM diagnosis is confirmed by observation of esophageal dilation on thoracic radiographs, with or without barium contrast [3,10] (Fig 1). Affected puppies fail to thrive and are at risk for aspiration pneumonia [11] and intussusception [12,13].

Neonatal mortality is high, but many CIM cases can be managed with a high-caloric liquid diet, frequent meals, and an elevated feeding regimen wherein dogs are held vertically to facilitate passage of food into the stomach [14]. Recently, administration of sildenafil was shown to ameliorate the clinical signs of both canine CIM and human idiopathic achalasia by promoting relaxation of the LES [15,16]. Most dogs with CIM require lifelong symptomatic management, but 20% to 46% of cases will spontaneously resolve by one year of age, suggesting that the disease may be attributed to delayed nerve development in the esophagus [3,17,18]. An esophagus-specific defect in afferent vagal innervation has been described in CIM-affected dogs [19,20].

CIM occurs across breeds, but the German shepherd dog (GSD) has the highest incidence, followed by Labrador retrievers, Great Danes, dachshunds, and miniature schnauzers [18,21–23]. We previously hypothesized that heritable factors underlie the high frequency of CIM in the GSD, and a preliminary study indicated a suggestive region of association on chromosome 12 and a complex pattern of inheritance [24]. We aim herein to conduct a robust genome-wide association study (GWAS) to identify genomic regions contributing to CIM and identify genetic variants that can be used as a tool to facilitate breeder efforts to reduce disease incidence.

## Results

### Study population

We recruited blood or buccal samples from 530 GSDs: 124 CIM-affected (70 males, 54 females) and 406 unaffected (165 males, 241 females) dogs (S1 Table). Samples were obtained primarily from two discrete United States populations: “pet GSDs” representing pets and other privately-owned dogs (108 affected, 303 unaffected) and “service GSDs” from breeding colonies maintained by multiple service organizations (16 affected, 103 unaffected). Genome-wide SNP genotypes were generated for 114 dogs (107 pet, 7 service). A principal component analysis showed no underlying population substructure (S1 Fig).

**Fig 2:**
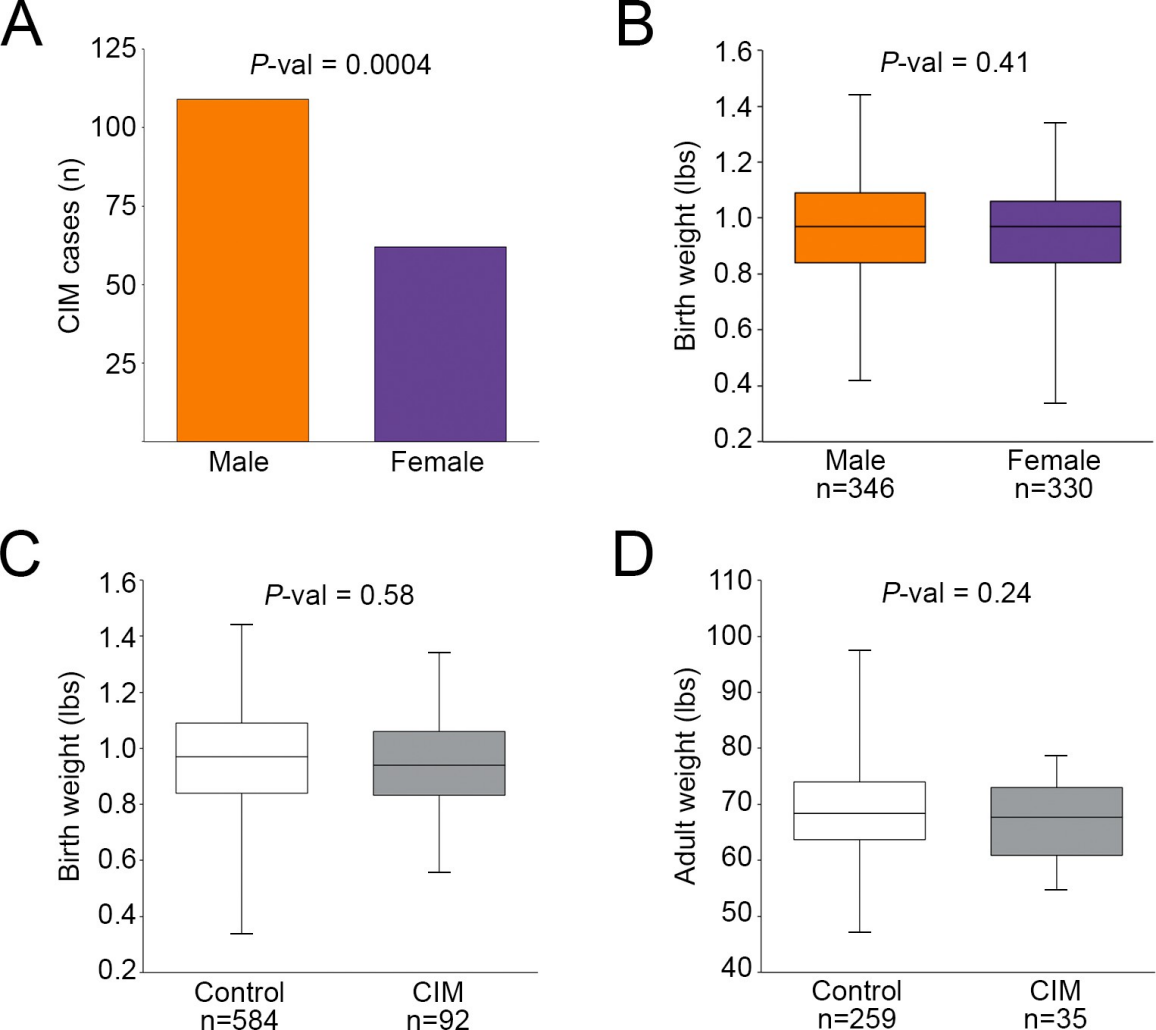
Phenotypic data from a private breeding colony. A) Bar graph of sex distribution in CIM cases shows a significant overrepresentation of males. Boxplots illustrate the absence of statistically significant differences in (B) birth weights between all males and females, (C) birth weights between affected and unaffected dogs, and (D) adult weights between affected and unaffected males.

Phenotypic records were obtained from 755 GSDs (16 are part of the aforementioned study population) from a service organization that maintains a private breeding colony (S2 Table). All dogs (affected and healthy) underwent barium studies at five weeks of age. In this larger cohort with stringent phenotyping, a significant proportion of cases were male (Fig 2A; 109 males, 62 females, *P*-val = 0.0004). Because GSD adult males are larger than females, we investigated whether body weight is correlated with CIM. Between the sexes, birth weights did not differ significantly (Fig 2B; 346 males, 330 females, *P*-val = 0.41). Weights of affected individuals were not significantly different from controls at birth (Fig 2C; 92 affected, 584 unaffected, *P*-val = 0.58), or at adulthood (Fig 2D; 35 affected males, 259 unaffected males, *P*-val = 0.24).

### Genome-wide association study

**Fig 3:**
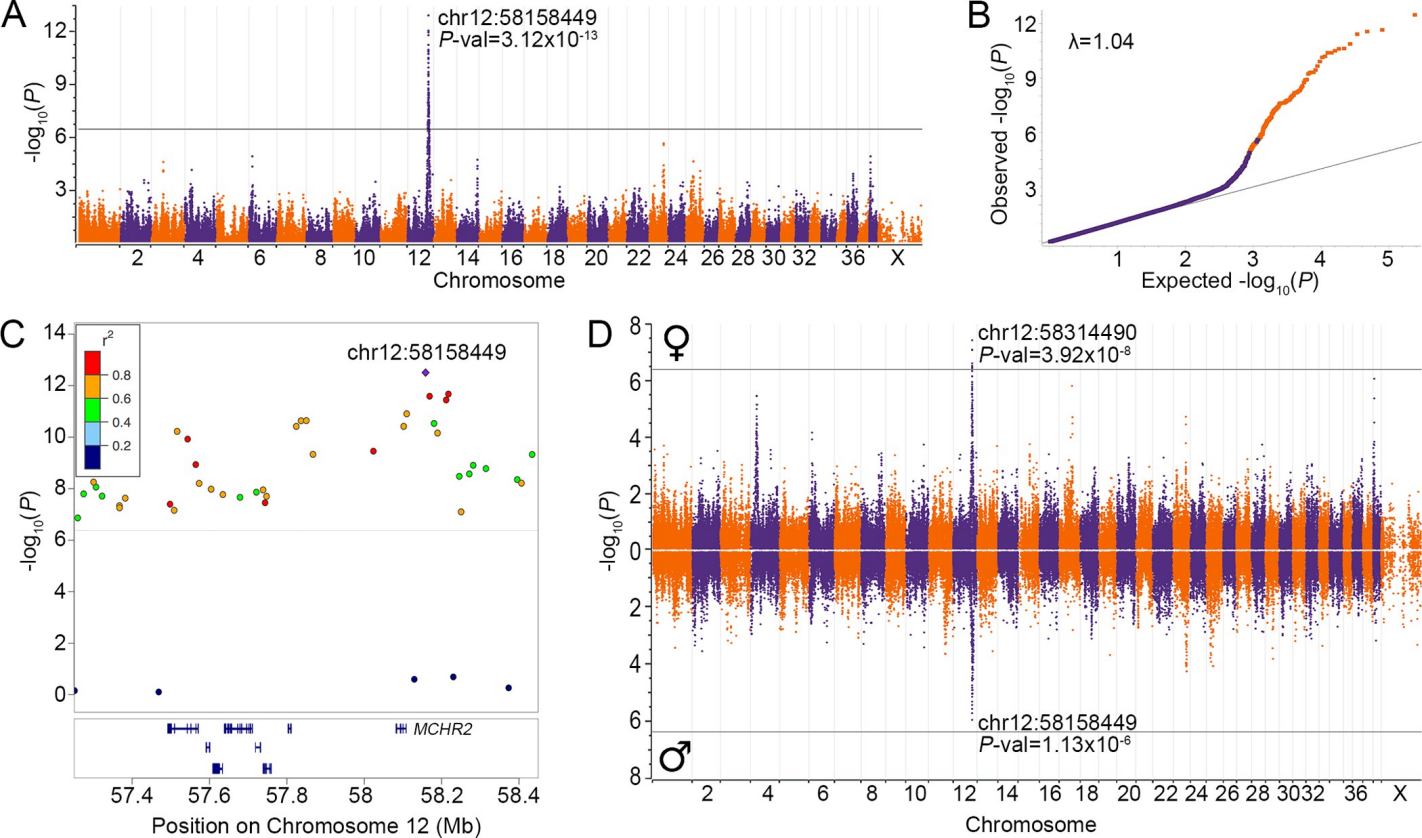
Genome-wide association results for CIM in GSDs. A) Manhattan plot of results from a GWAS for CIM using 59 affected and 53 control GSDs, with sex as a covariate. The –log_10_*P*-vals for 117,451 SNPs are plotted against genome position (CanFam3.1), with Bonferroni significance denoted by the black horizontal line. The *P*-val and position of the lead SNP are given. B) A Q-Q plot shows observed vs. expected –log_10_*P*-vals, with 454 SNPs within 5 Mb of the lead SNP indicated in orange. The genomic inflation factor (λ) is given. C) Regional chromosome 12 GWAS results are color-coded based on pairwise LD (r^2^) with the lead SNP (purple diamond), calculated using all 53 controls. The positions of protein-coding genes within the region are indicated by blue bars below, with *MCHR2* labeled. D) Miami plot of results from independent GWASs for CIM in females (top; 24 cases, 35 controls, λ = 1.042) and males (bottom; 35 cases, 18 controls, λ = 1.036).

We conducted a GWAS for CIM, with sex as a covariate, using 59 cases (24 female, 35 male), 53 controls (35 female, 18 male), and 117,451 SNPs, after filtering. A single region of association extending from 56.5 to 60 Mb on chromosome 12 includes 82 SNPs exceeding Bonferroni correction (*P*-val ≤ 4.26x10^-7^; Fig 3A and 3B). The lead SNP, chr12:58158449, has a *P*-val of 3.12x10^-13^ and R-squared of 0.43. High linkage disequilibrium (LD) with the lead SNP, defined by r^2^ >0.6, extends from 57.3 to 58.4 Mb and contains eight protein-coding genes (Fig 3C), none of which is known to underlie phenotypes in the dog.

To identify sex-specific loci contributing to CIM, we conducted independent GWASs in females (24 cases, 35 controls) and males (35 cases, 18 controls). Each GWAS yielded a primary signal on chromosome 12 (females: chr12:58314490, *P*-val = 3.92x10^-8^; males: chr12:58158449, *P*-val = 1.13x10^-6^) and neither revealed additional loci surpassing Bonferroni significance (Figs 3D and S2).

### Identification of candidate variants

Whole genome resequencing (WGS) data (ranging from 30 to 54X coverage) were generated for three, ancestrally diverse, affected female GSDs that were homozygous for the risk alleles of the leading 10 chromosome 12 SNPs (S3 Table). In the aforementioned 1.1 Mb region of high LD, 1,737 variants were homozygous in all three affected dog genomes. We used a variant call format (VCF) file containing WGS data from 1,330 domesticated dogs of pure and mixed breeds to evaluate non-structural variant allele frequencies. None of the 1,737 variants are unique to the affected dogs or the GSD breed. We generated structural VCF files and manually scanned the three affected GSD genomes in Integrative Genomics Viewer (IGV) to identify mobile elements and large deletions and insertions within the 1.1 Mb interval. All structural variants are present in multiple non-GSD genomes. No variants in the interval are predicted to impact protein sequence. Together, these results indicate that the CIM-associated variant is a non-coding polymorphism found across breeds.

**Fig 4:**
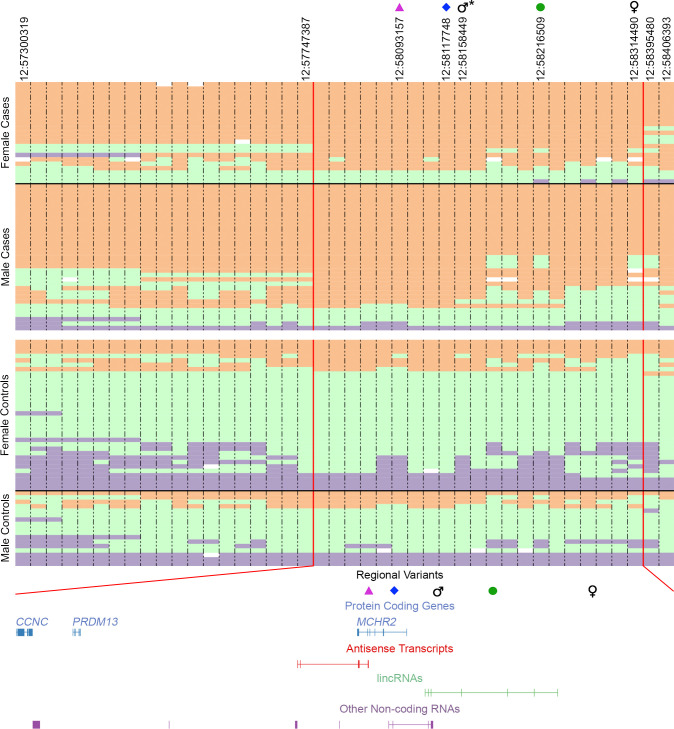
Haplotypes at the CIM-associated locus on chromosome 12. Genotypes from 23 female and 33 male cases (above the white line) and 34 female and 17 male controls are shown for the 1.1 Mb region of high LD. CIM-associated homozygous genotypes are orange while homozygous opposite genotypes are purple, heterozygous genotypes are light green, and missing genotypes are white. Uninformative SNPs were removed. Vertical red lines demarcate a 648 kb interval of high homozygosity among cases, defined by four heterozygous individuals to the centromeric end and three telomerically. CanFam3.1 positions are marked for the antisense transcript SNP (magenta triangle), VNTR (blue diamond), male GWAS lead SNP (male symbol), LINE insertion (green circle), and female GWAS lead SNP (female symbol). Genes and transcripts within the region of high homozygosity are shown below with variant positions noted. *The male GWAS shares the same lead SNP as the original GWAS with both sexes.

We observed that 85% of affected dogs from the GWAS were homozygous for the risk allele, compared to only 19% of controls, indicating that homozygosity for the chromosome 12 locus is a strong risk factor. We therefore delimited a narrower interval of 648 kb, wherein a maximum number of affected individuals are homozygous for the risk haplotype, defined by four heterozygous individuals to the centromeric end and three telomerically (Fig 4). Filtering to retain homozygous variants present in the three CIM genomes and absent from a publicly-available male GSD genome lacking the risk haplotype yielded 577 variants. Of these, 21 were in intronic or untranslated regions of *Melanin-Concentrating Hormone Receptor 2* (*MCHR2*, *ENSCAFG00000003533*.*5*), and the remaining were located intergenically to protein-coding genes and outside of promoter regions.

We next selected candidate variants for genotyping in a larger cohort to further assess their association with CIM. Within the 648 kb region of high homozygosity among cases (Fig 4), we identified three intronic variants in positions potentially impacting splicing or regulation of *MCHR2*: 1) a 4 bp deletion located 20 bp upstream of the exon six splice acceptor site, 2) a SNP in a transcribed region of an antisense transcript (*CFRNASEQ_AS_00025246*), and 3) a 33 bp variable number tandem repeat (VNTR) in a transcribed region of a non-coding transcript (*CFRNASEQ_IGNC_00025249*), upstream of the *MCHR2* translation initiation site. We also identified a compelling structural variant, a long-interspersed nuclear element (LINE) insertion, that lies within an intron of a lincRNA (*CFRNASEQ_IGNC_Spliced_00025252*) expressed in esophagus and brain. In the larger data set, the 4 bp deletion did not segregate with CIM, and the more distant LINE insertion was less significantly associated than either the antisense SNP or the VNTR. The latter two variants were similarly highly associated (S4 Table), with the differences in *P*-value appearing to be driven predominantly by genotypic changes among unaffected dogs.

**Fig 5:**
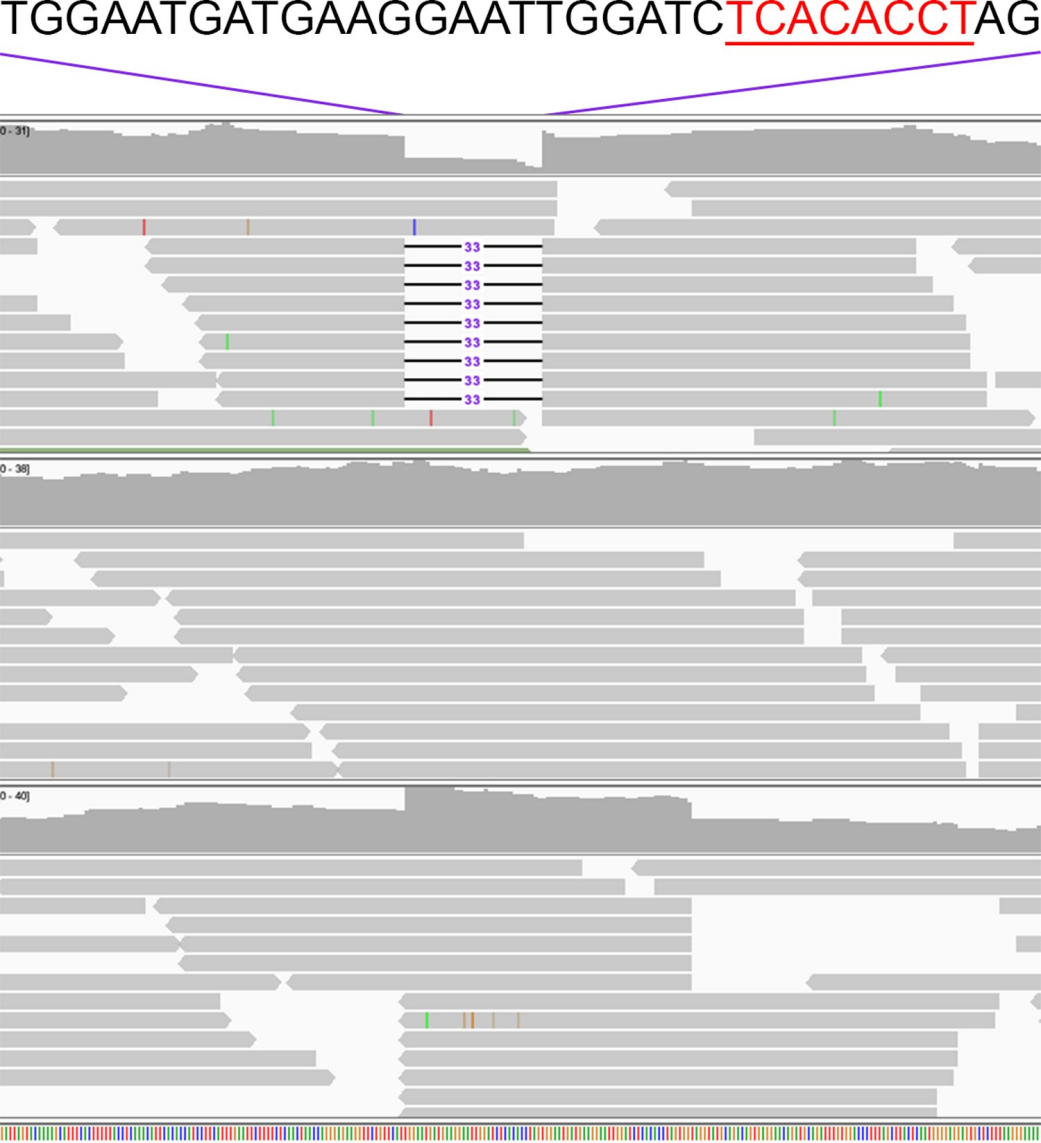
VNTR sequence and genotyping. Sequence of the 33 bp VNTR is shown, with the 8 bp T-box protein consensus sequence in underlined red text. IGV screenshot displays homozygosity for alleles *1* (top), *2* (middle; reference allele), and *3* (bottom) of the VNTR.

The antisense SNP is not well conserved evolutionarily across mammals, including those expressing *MCHR2*, and occurs in a transcript that is not annotated in the genomes of other species. Within the 33 bp VNTR are multiple predicted binding motifs, most notably an 8 bp T-half site (TCACACCT; *P*-val = 3.41x10^-6^ from TOMTOM) that matches the optimal consensus binding sequence for T-box family members (Fig 5) [25–28]. We focus the remainder of this study on the VNTR, although the complex inheritance of CIM and high regional LD prevent the exclusion of other linked variants as contributors to CIM.

### VNTR analysis

**Fig 6:**
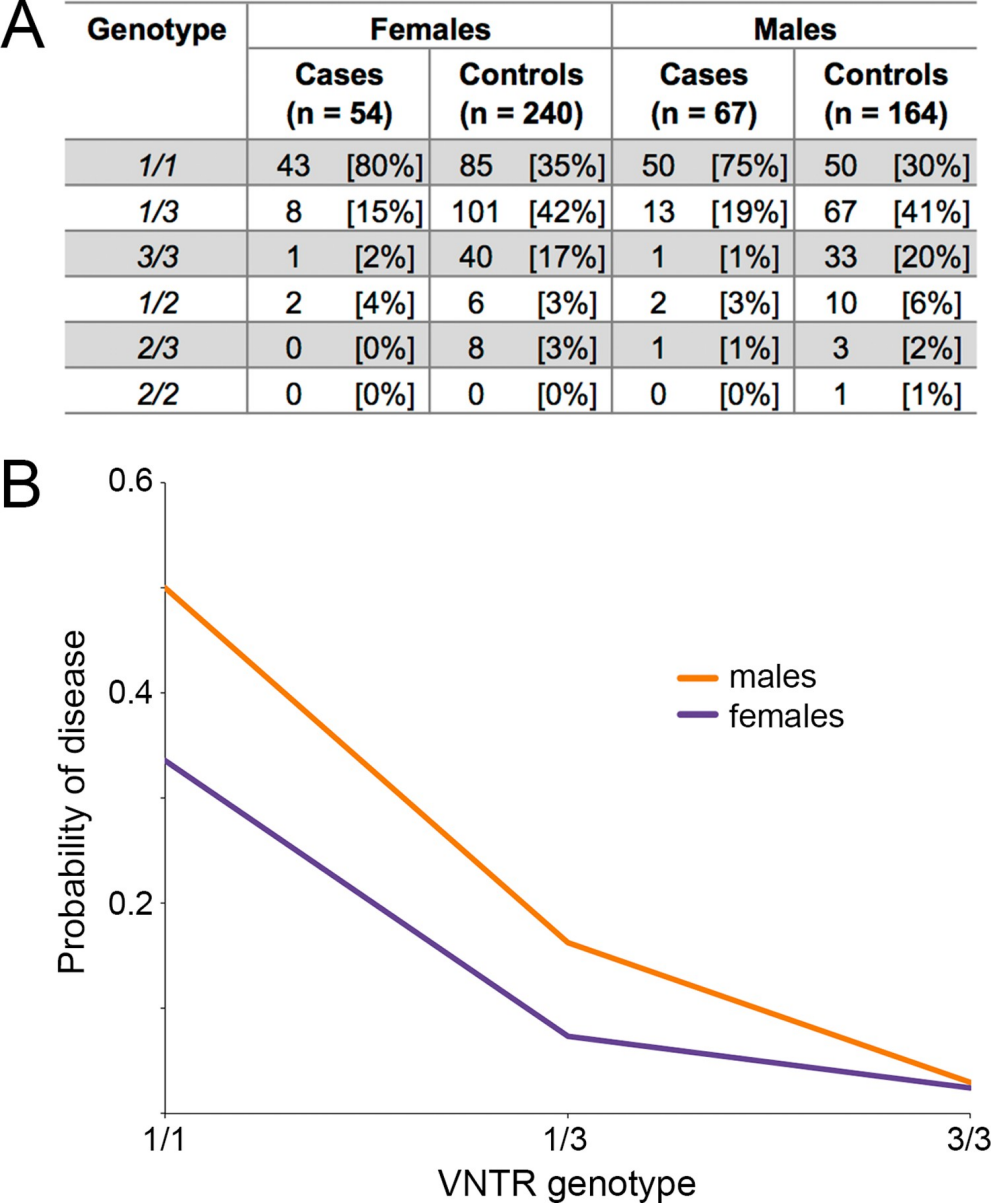
Observed VNTR genotypes and disease probabilities in GSDs. A) Observed numbers of cases and controls having each genotype are reported by sex in order of genotypic frequency. B) Probability of disease (y-axis) for the three most common genotypes (x-axis) are plotted for females and males. Probability of disease is significantly different between the sexes for the *1*/*1* (n = 228, *P*-val = 0.009) and *1*/*3* (n = 189, *P*-val = 0.046) genotypes.

To further evaluate the association of the VNTR, we genotyped an additional 58 CIM-affected (47 pet, 11 service) and 351 unaffected (253 pet, 98 service) GSDs. In the total VNTR genotyping population (n = 525), we detected three different alleles that we denote as *1*, *2*, or *3*, corresponding to the number of repeats of the 33 bp sequence (Fig 5 and S5 Table). The CanFam 3.1 reference genome has allele *2*. GSDs inherit two to six total copies of the repeat. Allele *1* is the major allele (56%) among healthy GSDs (n = 404), followed by alleles *3* and *2* (40% and 4%, respectively; Fig 6A). Allele *1* was strongly associated with CIM in the larger population (n = 492, *P*-val = 1.32x10^-17^; females: n = 278, *P*-val = 4.21x10^-9^; males: n = 214, *P*-val = 8.11x10^-11^; Fig 6A), with homozygosity for this allele conferring more significant risk (n = 303, *P*-val = 3.96x10^-10^) than heterozygosity (n = 264, *P*-val = 0.029). We observed significantly different probabilities of disease between the sexes, with *1*/*1* males having a 1.5X greater risk for disease compared to *1*/*1* females, and *1/3* males having a 2.2X greater risk than *1/3* females (Fig 6B).

**Fig 7:**
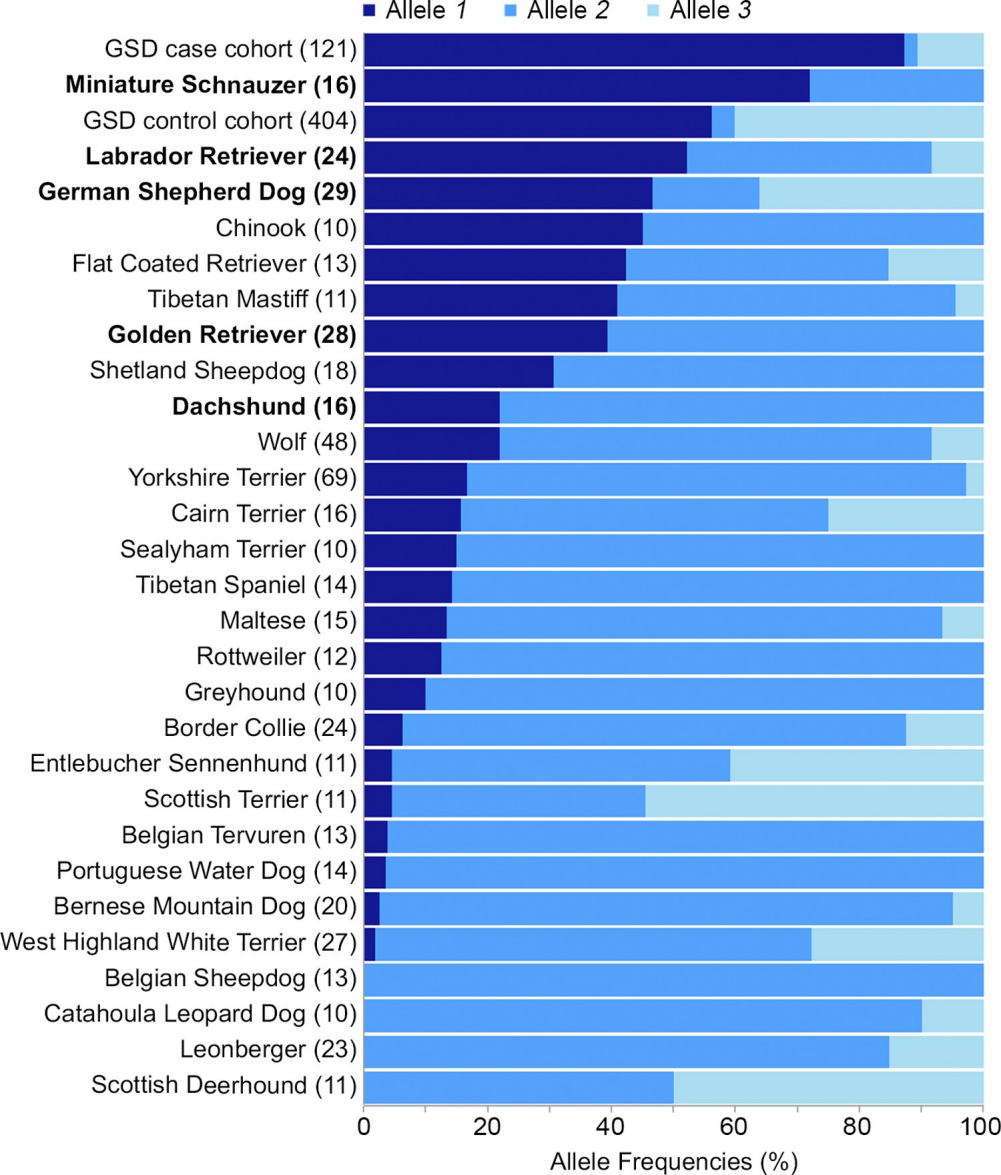
VNTR allele frequencies in dogs and wolves. Frequencies of alleles *1*, *2*, and *3* of the VNTR are given for our case and control cohorts, 27 dog breeds with VNTR genotypes from at least 10 individuals (including non-study GSDs from publicly available WGS data), and wolves. The number of individuals of each breed is shown in parentheses. Breeds having high incidences of CIM are bolded [23].

Across 1,323 dogs of pure and mixed breed ancestries, allele *2* is the major allele (73%) and alleles *1* and *3* have frequencies of 19% and 8%, respectively (Fig 7 and S6 Table). Allele *1* occurs in homozygosity in 9% of dogs and appears to be the major allele in Labrador retrievers and miniature schnauzers, both of which have high incidences of CIM (Fig 7). Among wolves (n = 48), *2* is the predominant allele (70%), followed by *1* (22%) and *3* (8%; Fig 7 and S6 Table). Three coyotes, one dhole, and one golden jackal have *2/2* genotypes, suggesting that allele *2* is the ancestral allele for canids, including the domestic dog (S6 Table). The VNTR is canid-specific; reference genomes of humans and other mammals that express *MCHR2* contain a single copy.

Together, the VNTR and sex predict disease with 77% accuracy. To identify additional loci involved in CIM, we conducted a second GWAS using the full cohort (59 cases, 53 controls) with sex and VNTR genotypes as covariates. No signals surpassed or approached Bonferroni significance (S3 Fig).

## Discussion

**O**ur study reveals a sex bias in CIM and a strong association with a VNTR in *MCHR2* on chromosome 12. *MCHR2* encodes one of two G-protein coupled receptors for melanin-concentrating hormone (MCH) [29,30], a neuropeptide synthesized in the region of the brain critical for feeding and reward [31,32]. MCH levels are directly correlated with food intake, weight, and gastrointestinal (GI) motility [33–38]. *MCH* is expressed across mammals, but *MCHR2* transcripts are only present in dogs, primates, and other higher order members [39,40]. Transgenic mice expressing human *MCHR2* have reduced food intake and body weight [40], whereas humans with deletions encompassing *MCHR2* and an adjacent gene, *Single-Minded Homolog 1* (*SIM1*), have increased appetite and obesity [41]. The *MCHR2/SIM1* locus also exerts sex-discordant effects on puberty timing, wherein the allele associated with earlier voice breaking in males is also linked to later onset of menarche in females [42].

Within intron one and upstream of the translational start site in exon two of *MCHR2*, we found a 33 bp canid-specific VNTR that contains a T-box binding consensus sequence known as a T-half site. The number of VNTR copies is inversely related to probability of CIM disease: dogs having six total copies are least likely to be affected whereas those with only two total copies have the highest disease incidence. The T-half site is bound by T-box transcription factors [25–28], and previous studies have illustrated that the number of T-half sites directly correlates with DNA binding [27]. T-box transcription factors can repress or activate target genes [43]. Future studies will be necessary to determine if *MCHR2* expression correlates with the number of T-half sites and if through this mechanism the VNTR influences MCH concentrations and GI motility.

GI motility is a key mediator of the sensations of hunger and fullness [44], with accelerated gastric emptying causing a shorter period of satiety and a stronger desire for food [45]. Feeding behaviors are under selection in dogs because food is commonly used as an incentive for positive behavior [46,47,48]. For example, a *pro-opiomelanocortin* (*POMC*) mutation associated with hunger and weight in Labrador retrievers has higher frequencies in service dog populations [46,47]. Neurons expressing *POMC* contribute to satiety signaling via regulation of GI motility [49], and it is worth noting that MCH mediates food intake through inhibition of POMC neuronal activity [50]. We posit that the number of VNTRs is directly related to GI motility: more repeats correlate with higher food motivation and protection from CIM, and fewer repeats correspond with reduced appetite and increased disease risk [51].

Our study reveals a significant sex bias in CIM. Females are affected less often than males and have lower penetrances of the VNTR risk genotypes. Although body size is a fundamental sexually-dimorphic trait, our data illustrate that CIM does not correlate with birth or adult weights. These findings hint at a female protective effect, wherein females have a biological advantage and therefore require a greater number of risk alleles, or genetic liability, to manifest CIM than do males [52].

The female sex hormone, estrogen, plays a role in increasing concentrations of the smooth muscle dilator nitric oxide (NO), which is the major neurotransmitter responsible for relaxing the LES [53,54]. Sex hormones are secreted before and after birth [55,56,57], thus they can impact the development of congenital disorders, like CIM. Higher female sex hormone levels have been linked to decreased LES pressure in pregnant women and postmenopausal women undergoing hormone replacement therapy [54]. Female dogs may have greater LES relaxation due to endogenous factors (*e*.*g*., higher estrogen levels), thereby facilitating the passage of food into the stomach and preventing the food retention that causes megaesophagus. We propose that in the absence of this protective effect, males are more susceptible to CIM. Our observations are consistent with male biases in human esophageal disorders, including reflux esophagitis and esophageal cancer, in which estrogen is thought to play a role [58].

In humans, the esophagus is comprised predominantly of smooth muscle, whereas in canids, nearly the entire length of the esophagus is striated muscle [59,60]. The LES is the only component of the canine esophagus dilated by NO [61]. Sildenafil, a drug widely used to treat CIM, reduces LES tone through the prevention of NO degradation [16]. NO levels also directly correlate with MCH levels [62], suggesting that a MCH imbalance may contribute to CIM status by impacting LES pressure.

In summary, we have uncovered a sex bias in CIM and a VNTR, intronic to *MCHR2*, that is strongly associated with CIM in GSDs. Together, sex and the VNTR predict greater than 75% of disease risk, but it is clear that there are additional factors influencing CIM in the breed. A genetic test is now available to help breeders increase the frequency of the low-risk allele *3*. Further studies are warranted to investigate the contribution of the VNTR and sex to CIM in other breeds, as well as gastric dilatation-volvulus (bloat), another GI motility disorder highly prevalent among GSDs [63].

## Materials and methods

### Ethics statement

All samples were obtained with informed consent according to protocols approved by the Clemson University Institutional Review Board (2013–18).

### Biologic sample population

Whole blood or buccal cells were obtained from 124 CIM-affected and 406 unaffected privately-owned and service GSDs from across the United States. All cases were diagnosed by a veterinarian via exclusion of non-idiopathic causes (*e*.*g*., persistent right aortic arch, myasthenia gravis) and a history of clinical signs from puppyhood, in conjunction with a standard or barium contrast radiograph. Pedigrees and radiographs were collected when available. Among GWAS cases, 95% of dogs were diagnosed at under one year of age. GWAS controls were over one year of age with no history of clinical signs consistent with CIM and no known relatives affected by CIM. Genomic DNA was isolated using the Gentra Puregene DNA Isolation kit (Qiagen). DNA concentration was quantitated by a NanoDrop 1000 spectrophotometer (Thermo Scientific).

### Phenotypic data population

Sex and CIM-affection data were collected from 755 affected and unaffected GSDs from a private breeding colony. All dogs underwent a barium swallow examination at five weeks of age. Birth and adult weight were obtained from subsets of 676 and 599 dogs, respectively.

### Genome-wide association and LD analyses

Individuals were selected for the association study such that sex and coat color were roughly balanced between cases and controls, and known relatives were excluded. Genome-wide SNP profiles were generated for 114 dogs (60 female, 54 male) using the Illumina CanineHD BeadChip, containing 220,853 SNPs (GeneSeek, Inc.). All filtering and statistical analyses were performed using SNP & Variation Suite v8 (SVS, Golden Helix) with chromosome positions in CanFam3.1. Two samples having call rates < 80% were pruned, as were 103,402 markers having < 95% call rates, minor allele frequencies < 0.05, and/or Hardy Weinberg Equilibrium *P*-values < 0.0004. Combined-sex GWASs for CIM were conducted with sex and VNTR genotypes as covariates, and *P*-values were calculated using a linear regression following a full vs. reduced model. All 53 controls were used in LD pairwise analyses between the lead SNP (chr12:58158449) and chromosome 12 SNPs, and plotted via LocusZoom [64]. Sex-specific GWASs were conducted using a linear regression following a full model and a marker set identical to the combined-sex GWASs. All chromosome positions are reported in CanFam3.1.

### Whole genome resequencing

Three affected GSDs were selected for WGS: a black/tan female with German ancestry (SRR15446412), a white female from the Netherlands (SRR15446416), and a black/tan female from an American service dog breeding colony (SRR15446414). Resequencing of genomes from the latter two dogs was performed using an Illumina HiSeq X Ten, generating 2x150 bp paired-end reads. Total reads generated ranged from 861 to 869 million per sample. Paired-end reads were trimmed, aligned to the indexed reference (CanFam3.1), sorted, and indexed to be viewed in IGV [65] using the Illumina DRAGEN (Dynamic Read Analysis for GENomics) Bio-IT platform [66]. WGS data for the third affected GSD were generated on an Illumina HiSeq 2000, with 2x125 bp paired-end reads. A total of 584 million reads were trimmed, aligned to CanFam3.1 with Bowtie2 [67], and sorted and indexed using SAMtools [68].

### Variant filtering

The DRAGEN pipeline was used to generate VCF files for two affected dogs, and SAMtools and BCFtools [69] were used to generate a VCF file for a third case. A VCF containing publicly-available WGS data from 1,330 dogs of pure and mixed breeds and 54 wild canids, generated following the methods described in [70], was used to assess allele frequencies in a broader canid population (S6 Table). Homozygous variants shared across the three affected dogs were selected using SVS. Within a 648 kb region of high homozygosity among cases (chr12:57,747,387–58,395,480), variants present in a male GSD lacking the risk haplotype (SRX4036121) were excluded from further analysis.

Structural VCF files were generated for the three affected GSDs and one GSD lacking the risk haplotype using SvABA [71]. The following settings for SvABA were used: ‘-r all’, ‘-k chr12:1–72,498,081’, and ‘-p 19’. The presence of alternate structural variants in other breeds was manually investigated in IGV using 15 genomes of nine other breeds (see Data Availability Statement).

Coding and splice site variants within predicted exons plus 50 bp flanking sequences were identified using CanFam3.1 Ensembl 89. Promoter and untranslated regions were defined in hg38 using GENCODEV36 and GeneHancer v5.4 and lifted over in the UCSC Genome Browser to CanFam3.1 positions. Transcription factor binding motifs were identified in TOMTOM [72].

### Genotyping

Primer sequences are given in S7 Table. The PCR for *CFRNASEQ_IGNC_Spliced_00025252* g.58216509_58216510ins(6444) used two forward primers, one upstream of and one within the LINE insertion, and a single downstream reverse primer. PCR for *MCHR2* g.58084223_58084226del was carried out using 2X ReddyMix (Thermo Scientific), and PCRs for *MCHR2* g.58093157T>A, *MCHR2* g.58117748_58117780del, chr12.g.58158449A>G, and *CFRNASEQ_IGNC_Spliced_00025252* g.58216509_58216510ins(6444) were carried out using *Taq* DNA Polymerase (Fisher BioReagents). *MCHR2* g.58117748_58117780del and *CFRNASEQ_IGNC_Spliced_00025252* g.58216509_58216510ins(6444) PCR products were run on a 3% agarose gel to determine genotypes by size (S4 Fig). Sanger sequencing (Eton Bioscience) was performed for the remaining variants using the BigDye Terminator v3.1 Cycle Sequencing Kit (Applied Biosystems) and an ABI 3730xl DNA Analyzer (Applied Biosystems). Three cases and two controls from the GWAS population were excluded from variant genotyping due to inadequate DNA quantities.

The VCF of 1,384 canid genomes was used to genotype *MCHR2* g.58117748_58117780del. At this positon, the reference allele contained two copies of the repeat and the alternate alleles were denoted as either a 33 bp deletion or insertion, corresponding to one or three copies of the repeat, respectively.

### Statistical analyses

Fisher’s exact two-tailed *P*-values were calculated to evaluate allelic and genotypic associations with CIM using VassarStats (http://vassarstats.net/). Because only 33 GSDs possessed the VNTR allele *2*, those individuals were excluded from the VNTR allelic association analysis. The genotypic associations of *1/1* and *1/3* with CIM were calculated using *3/3* dogs as a comparison. A one-way chi square test was used to assess the significance of male overrepresentation among cases (http://vassarstats.net/). Two-sample *t* tests were used to evaluate mean weight differences in males vs. females and affected vs. unaffected dogs. Probability of disease was calculated for each sex by dividing the number of cases having a particular genotype by the total number of dogs with that genotype. Fisher’s exact one-tailed *P*-values were calculated to assess the significance of disease probability differences between the sexes within the *1/1* and *1/3* genotypic groups (http://vassarstats.net/).

Our disease phenotype is binary, dogs are either affected or unaffected. The evaluation of such data is typically conducted with logistic regression, where we define the probability of disease for a dog of the *i*-th sex and *j*-th genotypic class as *p_ij_*. Accordingly, we define the logit of this probability as $\theta_{ij}=log[p_{ij}/(1-p_{ij})]$where the subsequent analysis is built with the following linear model:

$$\theta_{ij}=b_{0}+{sex}_{i}+{genotype}_{j}$$

where *b*_0_ is an unknown constant common to all dogs, *sex_i_* is the contribution of the *i*-th sex (*i* = F/M) and *genotype_j_* is the contribution of the *j*-th genotypic (*j* = 1/1, 1/2, 1/3, 2/3, 3/3) class. Estimation of the unknown effects and predictions of the risk of disease, are provided by the *glm* function of the public domain language R [73]. The accuracy of this model (and any additional sub-models) in the prediction of disease can be assessed through the receiver operating characteristic curve (using the area under the curve), fitted with the R package pROC [74].

### Dyad DOI

https://doi.org/10.5061/dryad.f7m0cfxz3 [75]

## Supporting information

S1 Fig Principal component analysis of the CIM combined-sex GWAS cohort (n = 112). Principal components 1 and 2 are plotted on the x- and y-axes, respectively.
(TIFF)

S2 Fig Q-Q plots of observed vs. expected –log_10_*P*-vals for GWASs for CIM in females (top) and males (bottom). The genomic inflation factors (λ) are given.
(TIFF)

S3 Fig Manhattan plot of GWAS results for CIM in GSDs (59 cases, 53 controls) with sex and VNTR genotypes as covariates. The –log_10_*P*-vals (y-axis) for 117,451 SNPs are plotted against chromosome position (x-axis). The threshold for Bonferroni significance is shown as a black horizontal line.
(TIFF)

S4 Fig Gel electrophoresis image of VNTR amplicons from dogs with various genotypes, shown above each lane. (TIFF)

S1 Table Phenotypic and genotypic data for 530 GSDs from the biologic sample population. (XLSX)

S2 Table Phenotypic data for 755 GSDs from a private breeding colony. (XLSX)

S3 Table Top 10 associated SNPs in GWAS for CIM. (XLSX)

S4 Table Candidate variants in chromosome 12 region of high LD. *The *MCHR2* g.58117748_58117780 reference allele includes two copies and alternate alleles have one (del) or three (dup) copies. Because the two-copy allele is uncommon in GSDs, individuals having two-copy alleles were excluded from the *MCHR2* g.58117748_58117780del statistics. For the remaining variants, A1 corresponds to the alternate allele and A2 to the reference allele.
(XLSX)

S5 Table Observed VNTR genotypes in service and pet GSDs. (XLSX)

S6 Table VNTR genotypes and accession numbers for 1,384 publicly available canid genomes. (XLSX)

S7 Table Primers for variant genotyping. (XLSX)

## References

[1] Nikaki K, Sawada A, Ustaoglu A, Sifrim D. Neuronal control of esophageal peristalsis and its role in esophageal disease. Current gastroenterology reports. 2019 Nov;21(11):1–9. doi: 10.1007/s11894-019-0728-z 31760496

[2] Hornby PJ, Abrahams TP. Central control of lower esophageal sphincter relaxation. The American journal of medicine. 2000 Mar 6;108(4):90–8. doi: 10.1016/s0002-9343(99)00345-9 10718459

[3] Johnson BM, DeNovo RC, Mears EA. Canine megaesophagus. Kirk’s Current Veterinary Therapy. 14th ed., Saunders Elsevier, St. Louis. 2009:486–92.

[4] Gockel HR, Schumacher J, Gockel I, Lang H, Haaf T, Nöthen MM. Achalasia: will genetic studies provide insights?. Human genetics. 2010 Oct;128(4):353–64. doi: 10.1007/s00439-010-0874-8 20700745

[5] Patti MG, Herbella FA. Achalasia and other esophageal motility disorders. Journal of Gastrointestinal Surgery. 2011 May;15(5):703–7. doi: 10.1007/s11605-011-1478-x 21394546

[6] Richter JE. High-resolution manometry in diagnosis and treatment of achalasia: help or hype. Current gastroenterology reports. 2014 Dec;16(12):1–7. doi: 10.1007/s11894-014-0420-2 25543338

[7] Bexfield NH, Watson PJ, Herrtage ME. Esophageal dysmotility in young dogs. Journal of veterinary internal medicine. 2006 Nov;20(6):1314–8. doi: 10.1892/0891-6640(2006)20[1314:ediyd]2.0.co;2 17186843

[8] Tams TR. Handbook of small animal gastroenterology. Elsevier Health Sciences; 2003.

[9] Palmer CS. Achalasia or cardiospasm in great Dane puppies. Veterinary medicine, small animal clinician: VM, SAC. 1968 Jun;63(6):574–6. 4385116

[10] McBrearty AR, Ramsey IK, Courcier EA, Mellor DJ, Bell R. Clinical factors associated with death before discharge and overall survival time in dogs with generalized megaesophagus. Journal of the American Veterinary Medical Association. 2011 Jun 15;238(12):1622–8. doi: 10.2460/javma.238.12.1622 21671818

[11] Kogan DA, Johnson LR, Sturges BK, Jandrey KE, Pollard RE. Etiology and clinical outcome in dogs with aspiration pneumonia: 88 cases (2004–2006). Journal of the American Veterinary Medical Association. 2008 Dec 1;233(11):1748–55. doi: 10.2460/javma.233.11.1748 19046034

[12] Graham KL, Buss MS, Dhein CR, Barbee DD, Seitz SE. Gastroesophageal intussusception in a Labrador retriever. The Canadian Veterinary Journal. 1998 Nov;39(11):709. 9818138 PMC1539471

[13] Grimes JA, Fleming JT, Singh A, Campbell BG, Hedlund CS, Tobias KM, et al. Characteristics and long-term outcomes of dogs with gastroesophageal intussusception. Journal of the American Veterinary Medical Association. 2020 Apr 15;256(8):914–20. doi: 10.2460/javma.256.8.914 32223709

[14] Haines JM, Khoo A, Brinkman E, Thomason JM, Mackin AJ. Technique for Evaluation of Gravity-Assisted Esophageal Transit Characteristics in Dogs with Megaesophagus. Journal of the American Animal Hospital Association. 2019;55(4):167–77. doi: 10.5326/JAAHA-MS-6711 31099601

[15] Bortolotti M, Mari C, Lopilato C, Porrazzo G, Miglioli M. Effects of sildenafil on esophageal motility of patients with idiopathic achalasia. Gastroenterology. 2000 Feb 1;118(2):253–7. doi: 10.1016/s0016-5085(00)70206-x 10648452

[16] Quintavalla F, Menozzi A, Pozzoli C, Poli E, Donati P, Wyler DK, et al. Sildenafil improves clinical signs and radiographic features in dogs with congenital idiopathic megaoesophagus: a randomised controlled trial. Veterinary Record. 2017 Apr;180(16):404–. doi: 10.1136/vr.103832 28188161

[17] Diamant N, Szczepanski M, Mui H. Idiopathic megaesophagus in the dog: reasons for spontaneous improvement and a possible method of medical therapy. The Canadian Veterinary Journal. 1974 Mar;15(3):66. 4831947 PMC1696374

[18] Cox VS, Wallace LJ, Anderson VE, Rushmer RA. Hereditary esophageal dysfunction in the Miniature Schnauzer dog. American journal of veterinary research. 1980 Mar 1;41(3):326–30. 7189391

[19] Holland CT, Satchell PM, Farrow BR. Vagal afferent dysfunction in naturally occurring canine esophageal motility disorder. Digestive diseases and sciences. 1994 Oct;39(10):2090–8. doi: 10.1007/BF02090355 7924726

[20] Holland CT, Satchell PM, Farrow BR. Selective vagal afferent dysfunction in dogs with congenital idiopathic megaoesophagus. Autonomic Neuroscience. 2002 Jul 31;99(1):18–23. doi: 10.1016/s1566-0702(02)00054-1 12171252

[21] Harvey CE, O’Brien JA, Durie VR, Miller DJ, Veenema R. Megaesophagus in the dog: a clinical survey of 79 cases. Journal of the American Veterinary Medical Association. 1974 Sep 1;165(5):443–6. 4420331

[22] Guilford WG. Megaesophagus in the dog and cat. InSeminars in veterinary medicine and surgery (small animal) 1990 (Vol. 5, No. 1, pp. 37–45). 2191392

[23] Haines JM. Survey of owners on population characteristics, diagnosis, and environmental, health, and disease associations in dogs with megaesophagus. Research in veterinary science. 2019 Apr 1;123:1–6. doi: 10.1016/j.rvsc.2018.11.026 30543946

[24] Tsai KL, Noorai RE, Starr-Moss AN, Quignon P, Rinz CJ, Ostrander EA, et al. Genome-wide association studies for multiple diseases of the German Shepherd Dog. Mammalian Genome. 2012 Feb;23(1):203–11. doi: 10.1007/s00335-011-9376-9 22105877 PMC3509149

[25] Kispert A, Herrmann BG. The Brachyury gene encodes a novel DNA binding protein. The EMBO journal. 1993 Aug;12(8):3211–20. 8344258 10.1002/j.1460-2075.1993.tb05990.x PMC413588

[26] Müller CW, Herrmann BG. Crystallographic structure of the T domain–DNA complex of the Brachyury transcription factor. Nature. 1997 Oct;389(6653):884–8. doi: 10.1038/39929 9349824

[27] Castellanos R, Xie Q, Zheng D, Cvekl A, Morrow BE. Mammalian TBX1 preferentially binds and regulates downstream targets via a tandem T-site repeat. PLoS One. 2014 May 5;9(5):e95151. doi: 10.1371/journal.pone.0095151 24797903 PMC4010391

[28] Baldini A, Fulcoli FG, Illingworth E. Tbx1: transcriptional and developmental functions. Current topics in developmental biology. 2017 Jan 1;122:223–43. doi: 10.1016/bs.ctdb.2016.08.002 28057265

[29] An S, Cutler G, Zhao JJ, Huang SG, Tian H, Li W, et al. Identification and characterization of a melanin-concentrating hormone receptor. Proceedings of the National Academy of Sciences. 2001 Jun 19;98(13):7576–81. 10.1073/pnas.131200698 PMC34710 11416225

[30] Sailer AW, Sano H, Zeng Z, McDonald TP, Pan J, Pong SS, et al. Identification and characterization of a second melanin-concentrating hormone receptor, MCH-2R. Proceedings of the National Academy of Sciences. 2001 Jun 19;98(13):7564–9. doi: 10.1073/pnas.121170598 11404457 PMC34708

[31] Gao XB. Electrophysiological effects of MCH on neurons in the hypothalamus. Peptides. 2009 Nov 1;30(11):2025–30. doi: 10.1016/j.peptides.2009.05.006 19463877 PMC2782585

[32] Stuber GD, Wise RA. Lateral hypothalamic circuits for feeding and reward. Nature neuroscience. 2016 Feb;19(2):198–205. doi: 10.1038/nn.4220 26814589 PMC4927193

[33] Qu D, Ludwig DS, Gammeltoft S, Piper M, Pelleymounter MA, Cullen MJ, et al. A role for melanin-concentrating hormone in the central regulation of feeding behaviour. Nature. 1996 Mar;380(6571):243–7. doi: 10.1038/380243a0 8637571

[34] Shimada M, Tritos NA, Lowell BB, Flier JS, Maratos-Flier E. Mice lacking melanin-concentrating hormone are hypophagic and lean. Nature. 1998 Dec;396(6712):670–4. doi: 10.1038/25341 9872314

[35] Della-Zuana O, Presse F, Ortola C, Duhault J, Nahon JL, Levens N. Acute and chronic administration of melanin-concentrating hormone enhances food intake and body weight in Wistar and Sprague–Dawley rats. International journal of obesity. 2002 Oct;26(10):1289–95. doi: 10.1038/sj.ijo.0802079 12355323

[36] Gomori A, Ishihara A, Ito M, Mashiko S, Matsushita H, Yumoto M, et al. Chronic intracerebroventricular infusion of MCH causes obesity in mice. American Journal of Physiology-Endocrinology and Metabolism. 2003 Mar 1;284(3):E583–8. doi: 10.1152/ajpendo.00350.2002 12453827

[37] Kokkotou E, Jeon JY, Wang X, Marino FE, Carlson M, Trombly DJ, et al. Mice with MCH ablation resist diet-induced obesity through strain-specific mechanisms. American Journal of Physiology-Regulatory, Integrative and Comparative Physiology. 2005 Jul;289(1):R117–24. doi: 10.1152/ajpregu.00861.2004 15731402

[38] Xu L, Wang H, Gong Y, Pang M, Sun X, Guo F, et al. Nesfatin-1 regulates the lateral hypothalamic area melanin-concentrating hormone-responsive gastric distension-sensitive neurons and gastric function via arcuate nucleus innervation. Metabolism. 2017 Feb 1;67:14–25. doi: 10.1016/j.metabol.2016.10.010 28081774

[39] Tan CP, Sano H, Iwaasa H, Pan J, Sailer AW, Hreniuk DL, et al. Melanin-concentrating hormone receptor subtypes 1 and 2: species-specific gene expression. Genomics. 2002 Jun 1;79(6):785–92. doi: 10.1006/geno.2002.6771 12036292

[40] Chee MJ, Pissios P, Prasad D, Maratos-Flier E. Expression of melanin-concentrating hormone receptor 2 protects against diet-induced obesity in male mice. Endocrinology. 2014 Jan 1;155(1):81–8. doi: 10.1210/en.2013-1738 24169555 PMC3868808

[41] El Khattabi L, Guimiot F, Pipiras E, Andrieux J, Baumann C, Bouquillon S, et al. Incomplete penetrance and phenotypic variability of 6q16 deletions including SIM1. European Journal of Human Genetics. 2015 Aug;23(8):1010–8. doi: 10.1038/ejhg.2014.230 25351778 PMC4795105

[42] Day FR, Bulik-Sullivan B, Hinds DA, Finucane HK, Murabito JM, Tung JY, et al. Shared genetic aetiology of puberty timing between sexes and with health-related outcomes. Nature communications. 2015 Nov 9;6(1):1–6. doi: 10.1038/ncomms9842 26548314 PMC4667609

[43] Papaioannou VE. The T-box gene family: emerging roles in development, stem cells and cancer. Development. 2014 Oct 15;141(20):3819–33. doi: 10.1242/dev.104471 25294936 PMC4197708

[44] Xing J, Chen JD. Alterations of gastrointestinal motility in obesity. Obesity research. 2004 Nov;12(11):1723–32. doi: 10.1038/oby.2004.213 15601965

[45] Halawi H, Camilleri M, Acosta A, Vazquez-Roque M, Oduyebo I, Burton D, et al. Relationship of gastric emptying or accommodation with satiation, satiety, and postprandial symptoms in health. American Journal of Physiology-Gastrointestinal and Liver Physiology. 2017 Nov 1;313(5):G442–7. doi: 10.1152/ajpgi.00190.2017 28774870 PMC5792209

[46] Raffan E, Dennis RJ, O’Donovan CJ, Becker JM, Scott RA, Smith SP, et al. A deletion in the canine POMC gene is associated with weight and appetite in obesity-prone labrador retriever dogs. Cell metabolism. 2016 May 10;23(5):893–900. doi: 10.1016/j.cmet.2016.04.012 27157046 PMC4873617

[47] Yeo GS. Genetics of obesity: can an old dog teach us new tricks?. Diabetologia. 2017 May;60(5):778–83. doi: 10.1007/s00125-016-4187-x 28013339 PMC6518377

[48] LaFollette MR, Rodriguez KE, Ogata N, O’Haire ME. Military veterans and their PTSD service dogs: associations between training methods, PTSD severity, dog behavior, and the human-animal bond. Frontiers in veterinary science. 2019 Feb 11;6:23. doi: 10.3389/fvets.2019.00023 30805353 PMC6378910

[49] Guan X, Shi X, Li X, Chang B, Wang Y, Li D, et al. GLP-2 receptor in POMC neurons suppresses feeding behavior and gastric motility. American Journal of Physiology-Endocrinology and Metabolism. 2012 Oct 1;303(7):E853–64. doi: 10.1152/ajpendo.00245.2012 22829581 PMC3469617

[50] Al-Massadi O, Quiñones M, Clasadonte J, Hernandez-Bautista R, Romero-Picó A, Folgueira C, et al. MCH regulates SIRT1/FoxO1 and reduces POMC neuronal activity to induce hyperphagia, adiposity, and glucose intolerance. Diabetes. 2019 Dec 1;68(12):2210–22. doi: 10.2337/db19-0029 31530579 PMC6868473

[51] Diniz GB, Bittencourt JC. The melanin-concentrating hormone as an integrative peptide driving motivated behaviors. Frontiers in systems neuroscience. 2017 May 29;11:32. doi: 10.3389/fnsys.2017.00032 28611599 PMC5447028

[52] Khramtsova EA, Davis LK, Stranger BE. The role of sex in the genomics of human complex traits. Nature Reviews Genetics. 2019 Mar;20(3):173–90. doi: 10.1038/s41576-018-0083-1 30581192

[53] Weiner CP, Lizasoain I, Baylis SA, Knowles RG, Charles IG, Moncada S. Induction of calcium-dependent nitric oxide synthases by sex hormones. Proceedings of the National Academy of Sciences. 1994 May 24;91(11):5212–6. doi: 10.1073/pnas.91.11.5212 7515189 PMC43962

[54] Nordenstedt H, Zheng Z, Cameron AJ, Ye W, Pedersen NL, Lagergren J. Postmenopausal hormone therapy as a risk factor for gastroesophageal reflux symptoms among female twins. Gastroenterology. 2008 Apr 1;134(4):921–8. doi: 10.1053/j.gastro.2008.01.009 18294635 PMC2359826

[55] Robinson JD, Judd HL, Young PE, Jones OW, Yen SS. Amniotic fluid androgens and estrogens in midgestation. The Journal of Clinical Endocrinology & Metabolism. 1977 Oct 1;45(4):755–61. doi: 10.1210/jcem-45-4-755 144143

[56] van de Beek C, van Goozen SH, Buitelaar JK, Cohen-Kettenis PT. Prenatal sex hormones (maternal and amniotic fluid) and gender-related play behavior in 13-month-old infants. Archives of Sexual Behavior. 2009 Feb;38(1):6–15. doi: 10.1007/s10508-007-9291-z 18080735

[57] Lanciotti L, Cofini M, Leonardi A, Penta L, Esposito S. Up-to-date review about minipuberty and overview on hypothalamic-pituitary-gonadal axis activation in fetal and neonatal life. Frontiers in endocrinology. 2018 Jul 23;9:410. doi: 10.3389/fendo.2018.00410 30093882 PMC6070773

[58] Chen C, Gong X, Yang X, Shang X, Du Q, Liao Q, et al. The roles of estrogen and estrogen receptors in gastrointestinal disease. Oncology letters. 2019 Dec 1;18(6):5673–80. doi: 10.3892/ol.2019.10983 31788039 PMC6865762

[59] Shiina T, Shimizu Y, Izumi N, Suzuki Y, Asano M, Atoji Y, et al. A comparative histological study on the distribution of striated and smooth muscles and glands in the esophagus of wild birds and mammals. Journal of veterinary medical science. 2005;67(1):115–7 10.1292/jvms.67.115 15699607

[60] Kallmünzer B, Sörensen B, Neuhuber WL, Wörl J. Enteric co-innervation of striated muscle fibres in human oesophagus. Neurogastroenterology & Motility. 2008 Jun;20(6):597–610. doi: 10.1111/j.1365-2982.2007.01075.x 18221249

[61] Wallace JL. Nitric oxide in the gastrointestinal tract: opportunities for drug development. British journal of pharmacology. 2019 Jan;176(2):147–54. doi: 10.1111/bph.14527 30357812 PMC6295408

[62] Varas M, Pérez M, Monzón ME, de Barioglio SR. Melanin-concentrating hormone, hippocampal nitric oxide levels and memory retention. Peptides. 2002 Dec 1;23(12):2213–21. doi: 10.1016/s0196-9781(02)00252-8 12535701

[63] Bell JS. Inherited and predisposing factors in the development of gastric dilatation volvulus in dogs. Topics in companion animal medicine. 2014 Sep 1;29(3):60–3. doi: 10.1053/j.tcam.2014.09.002 25496921

[64] Pruim RJ, Welch RP, Sanna S, Teslovich TM, Chines PS, Gliedt TP, et al. LocusZoom: regional visualization of genome-wide association scan results. Bioinformatics. 2010 Sep 15;26(18):2336–7. doi: 10.1093/bioinformatics/btq419 20634204 PMC2935401

[65] Thorvaldsdóttir H, Robinson JT, Mesirov JP. Integrative Genomics Viewer (IGV): high-performance genomics data visualization and exploration. Briefings in bioinformatics. 2013 Mar 1;14(2):178–92. doi: 10.1093/bib/bbs017 22517427 PMC3603213

[66] Miller NA, Farrow EG, Gibson M, Willig LK, Twist G, Yoo B, et al. A 26-hour system of highly sensitive whole genome sequencing for emergency management of genetic diseases. Genome medicine. 2015 Dec;7(1):1–6. doi: 10.1186/s13073-014-0122-2 26419432 PMC4588251

[67] Langmead B, Salzberg SL. Fast gapped-read alignment with Bowtie 2. Nature methods. 2012 Apr;9(4):357–9. doi: 10.1038/nmeth.1923 22388286 PMC3322381

[68] Li H, Handsaker B, Wysoker A, Fennell T, Ruan J, Homer N, et al. The sequence alignment/map format and SAMtools. Bioinformatics. 2009 Aug 15;25(16):2078–9. doi: 10.1093/bioinformatics/btp352 19505943 PMC2723002

[69] Li H. A statistical framework for SNP calling, mutation discovery, association mapping and population genetical parameter estimation from sequencing data. Bioinformatics. 2011 Nov 1;27(21):2987–93. doi: 10.1093/bioinformatics/btr509 21903627 PMC3198575

[70] Plassais J, Kim J, Davis BW, Karyadi DM, Hogan AN, Harris AC, et al. Whole genome sequencing of canids reveals genomic regions under selection and variants influencing morphology. Nature communications. 2019 Apr 2;10(1):1–4. doi: 10.1038/s41467-018-07882-8 30940804 PMC6445083

[71] Wala JA, Bandopadhayay P, Greenwald NF, O’Rourke R, Sharpe T, Stewart C, et al. SvABA: genome-wide detection of structural variants and indels by local assembly. Genome research. 2018 Apr 1;28(4):581–91. doi: 10.1101/gr.221028.117 29535149 PMC5880247

[72] Gupta S, Stamatoyannopoulos JA, Bailey TL, Noble WS. Quantifying similarity between motifs. Genome biology. 2007 Feb;8(2):1–9. doi: 10.1186/gb-2007-8-2-r24 17324271 PMC1852410

[73] Team RC. R: A language and environment for statistical computing. [Internet]. Vienna, Austria: R Foundation for Statistical Computing; 2021. Available from: http://www.r-project.org

[74] Robin X, Turck N, Hainard A, Tiberti N, Lisacek F, Sanchez JC, et al. pROC: an open-source package for R and S+ to analyze and compare ROC curves. BMC bioinformatics. 2011 Dec;12(1):1–8. doi: 10.1186/1471-2105-12-77 21414208 PMC3068975

[75] Clark LA et al. (2021) Data from: SNP genotypes for healthy and CIM-affected GSDs. Dryad Digital Repository. Openly available via 10.5061/dryad.f7m0cfxz3

