# Supplementary figures and images for: Congenital idiopathic megaesophagus in the German shepherd dog is a sex-differentiated trait and is associated with an intronic variable number tandem repeat in Melanin-Concentrating Hormone Receptor 2
Source: PLoS Genet. 2022 Mar 10;18(3):e1010044. doi: 10.1371/journal.pgen.1010044 (PMC8912139; doi:10.1371/journal.pgen.1010044)

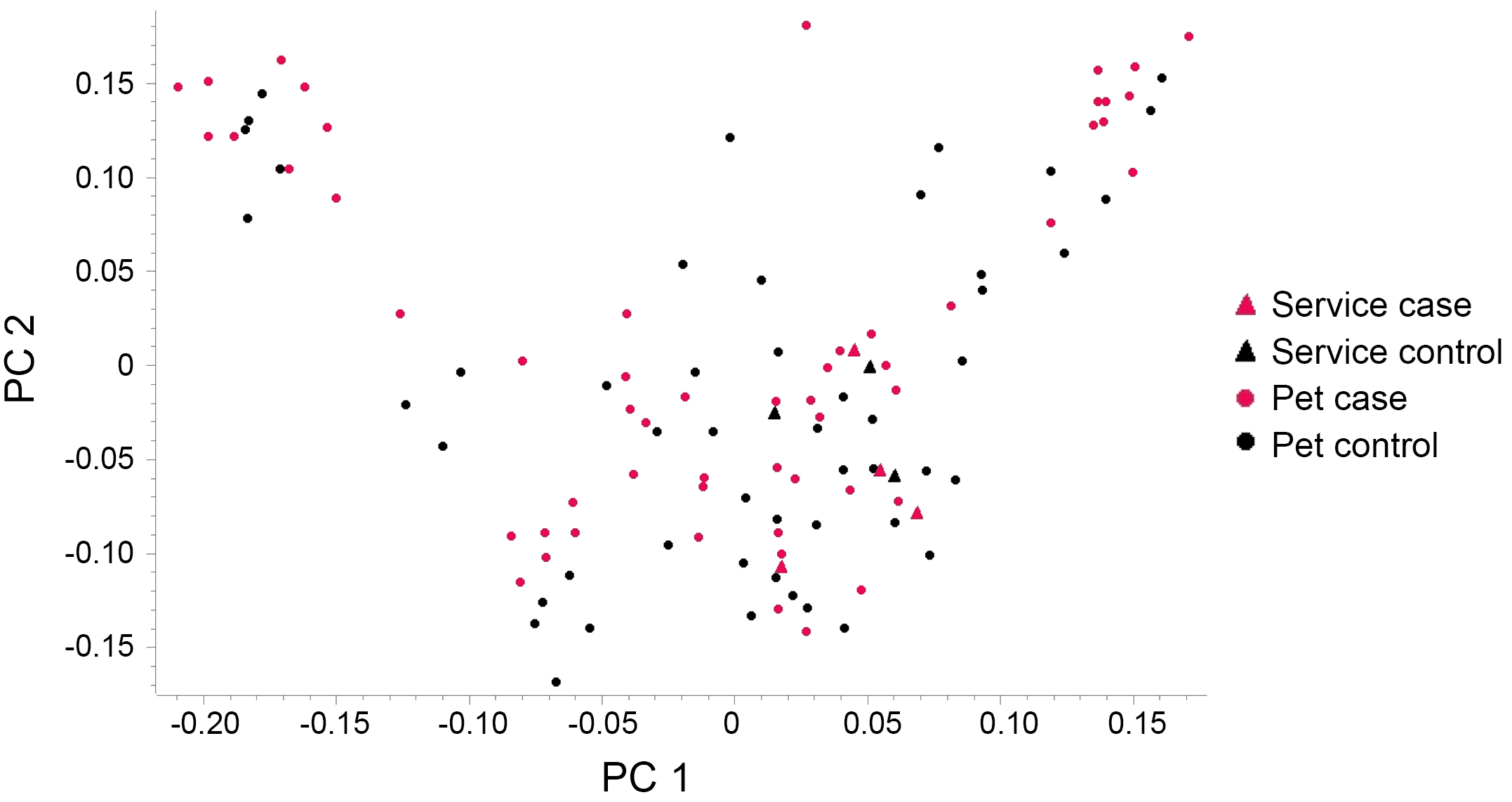

Supplement: S1 Fig — Principal components 1 and 2 are plotted on the x- and y-axes, respectively. (TIFF) [file pgen.1010044.s001.tiff]

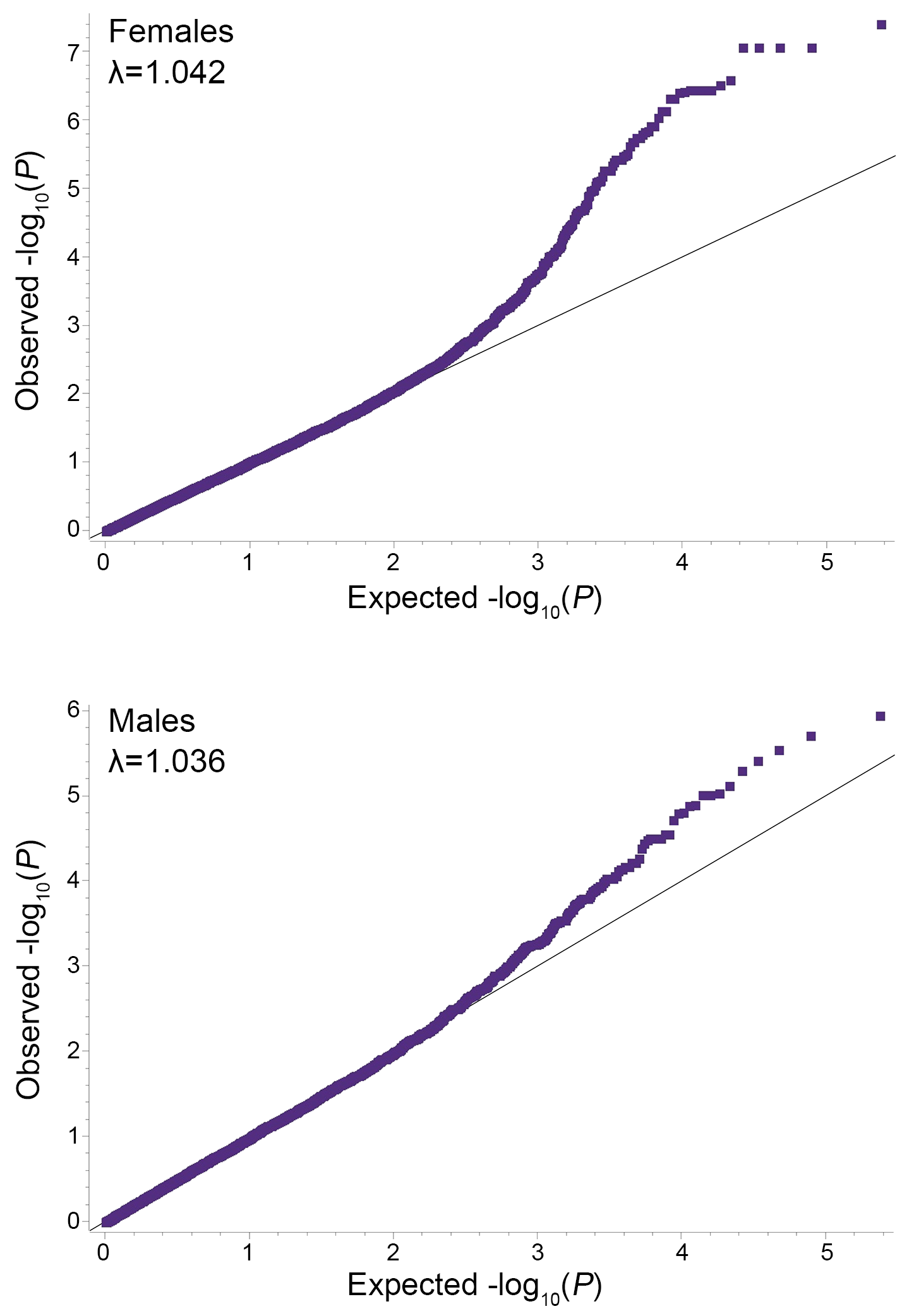

Supplement: S2 Fig — The genomic inflation factors (λ) are given. (TIFF) [file pgen.1010044.s002.tiff]

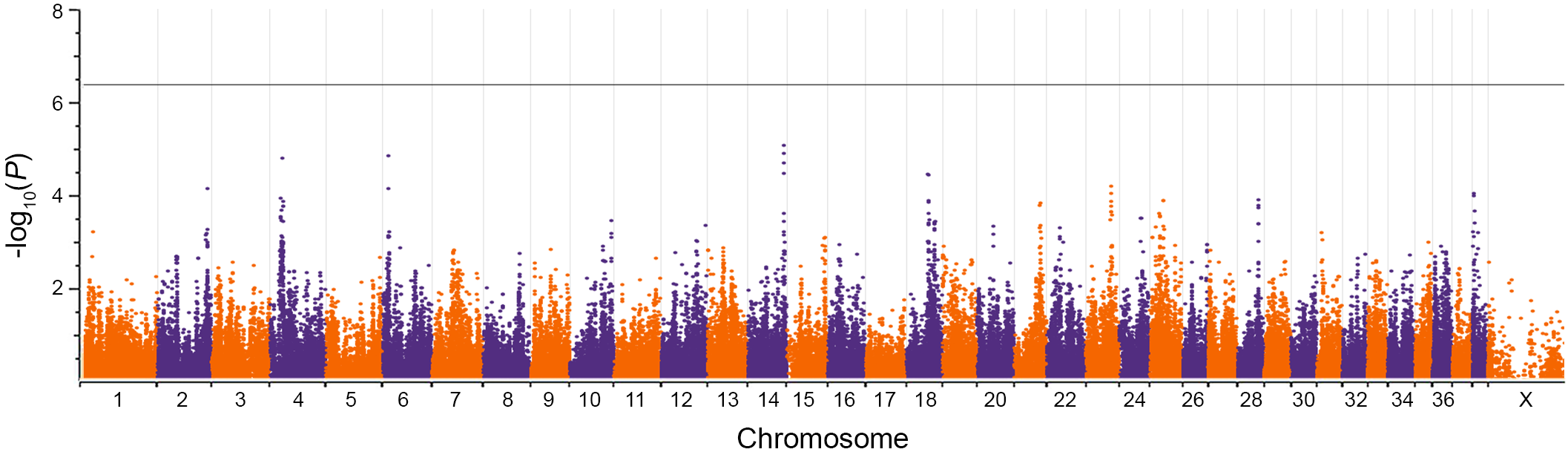

Supplement: S3 Fig — The –log10P-vals (y-axis) for 117,451 SNPs are plotted against chromosome position (x-axis). The threshold for Bonferroni significance is shown as a black horizontal line. (TIFF) [file pgen.1010044.s003.tiff]

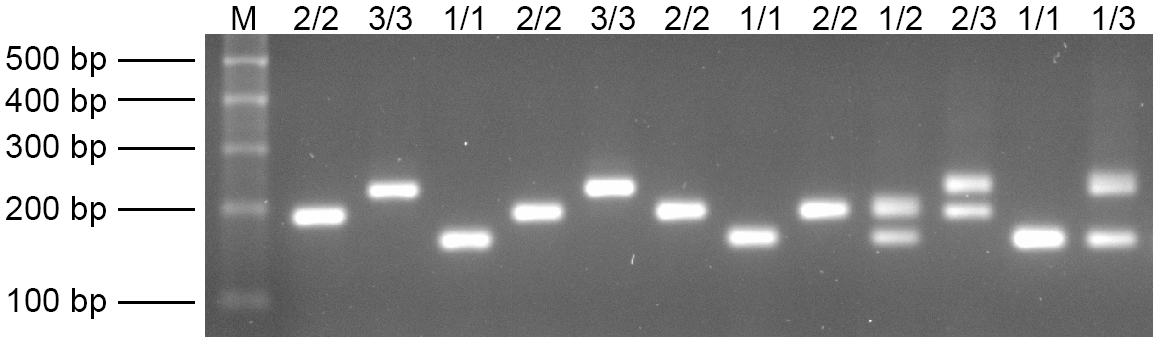

Supplement: S4 Fig — (TIFF) [file pgen.1010044.s004.tiff]
